# CONNECTING THE DOTS: CHRONIC CARE MANAGEMENT PROGRAM IMPLEMENTATION IN RURAL PRACTICES

**DOI:** 10.1093/geroni/igac059.888

**Published:** 2022-12-20

**Authors:** Faith Jones, Kevin Franke, Tonja Woods, Emma Bjore, Elizabeth Punke

**Affiliations:** HealthTech, Brentwood, Tennessee, United States; University of Wyoming, Laramie, Wyoming, United States; University of Wyoming, Laramie, Wyoming, United States; Ivinson Medical Group, Laramie, Wyoming, United States; University Of Wyoming, Laramie, Wyoming, United States

## Abstract

As the focus of healthcare changes from a “sick” care model to a population health model, primary care and specialty clinic practices have new opportunities supported through Medicare reimbursement. The incorporation of team-based care coordination programs into clinic practices is an important step towards value-based care and achieving the Triple Aim: better health for the population, better care for individuals, and lower costs through improvements. Since 2019, six rural Wyoming primary care practices have completed training to implement and expand care coordination programs. HealthTechS3 provides participating clinics with team-based training in the implementation of the Chronic Care Management Program, Behavioral Health Integration, and other billable care coordination services. To date, 301 patients have enrolled in care coordination services. These practices have generated $350,000 in revenue. Using a consolidated implementation framework as a guide, critical components of successful rural care coordination program implementation are discussed.

